# A *cis*-Acting Mutation in the *PxABCG1* Promoter Is Associated with Cry1Ac Resistance in *Plutella xylostella* (L.)

**DOI:** 10.3390/ijms22116106

**Published:** 2021-06-05

**Authors:** Jianying Qin, Fan Ye, Linzheng Xu, Xuguo Zhou, Neil Crickmore, Xiaomao Zhou, Youjun Zhang, Zhaojiang Guo

**Affiliations:** 1 Longping Branch, Graduate School of Hunan University, Changsha 410125, China; qinjianying0203@163.com (J.Q.); zhouxm1972@126.com (X.Z.); 2 Department of Plant Protection, Institute of Vegetables and Flowers, Chinese Academy of Agricultural Sciences, Beijing 100081, China; yefan9605@163.com (F.Y.); xulinzheng1112@163.com (L.X.); zhangyoujun@caas.cn (Y.Z.); 3 Department of Entomology, University of Kentucky, Lexington, KY 40546-0091, USA; xuguozhou@uky.edu; 4 School of Life Sciences, University of Sussex, Brighton BN1 9QG, UK; n.crickmore@sussex.ac.uk

**Keywords:** *Bacillus thuringiensis*, *Plutella xylostella*, *cis*-mutation, Antp, ABCG1, Cry1Ac resistance

## Abstract

The molecular mechanisms of insect resistance to Cry toxins generated from the bacterium *Bacillus thuringiensis* (Bt) urgently need to be elucidated to enable the improvement and sustainability of Bt-based products. Although downregulation of the expression of midgut receptor genes is a pivotal mechanism of insect resistance to Bt Cry toxins, the underlying transcriptional regulation of these genes remains elusive. Herein, we unraveled the regulatory mechanism of the downregulation of the ABC transporter gene *PxABCG1* (also called *Pxwhite*), a functional midgut receptor of the Bt Cry1Ac toxin in *Plutella xylostella.* The *PxABCG1* promoters of Cry1Ac-susceptible and Cry1Ac-resistant strains were cloned and analyzed, and they showed clear differences in activity. Subsequently, a dual-luciferase reporter assay, a yeast one-hybrid (Y1H) assay, and RNA interference (RNAi) experiments demonstrated that a *cis*-mutation in a binding site of the Hox transcription factor Antennapedia (Antp) decreased the promoter activity of the resistant strain and eliminated the binding and regulation of Antp, thereby enhancing the resistance of *P. xylostella* to the Cry1Ac toxin. These results advance our knowledge of the roles of *cis*- and *trans*-regulatory variations in the regulation of midgut Cry receptor genes and the evolution of Bt resistance, contributing to a more complete understanding of the Bt resistance mechanism.

## 1. Introduction

Insecticidal proteins from the gram-positive bacterium *Bacillus thuringiensis* (Bt) have become the most successful alternatives to chemical pesticides because of their efficient and specific insecticidal activity and their environmental benignity [1,2,3,4]. To date, biopesticides and transgenic crops based on recombinant Bt or Bt toxins have been widely used for pest control worldwide, making substantial contributions to socioeconomic development and environmental sustainability [5]. Unfortunately, the benefits and long-term application potential of Bt products are severely threatened by evolved resistance in insects [6,7]. Therefore, clarifying the molecular mechanisms of Bt resistance is essential for delaying the evolution of insect resistance to Bt Cry toxins and for sustainably utilizing Bt products.

The Bt Cry proteins exert their toxicity through multiple main steps in the larval midgut, and the interaction of Cry toxins with functional receptors is critical for their cytotoxicity, and post-binding events result in cell lysis and death [4,8,9,10]. Empirical evidence demonstrates that the functional receptors for Bt toxins in the midgut include cadherin (CAD), alkaline phosphatase (ALP), aminopeptidase N (APN), and ATP-binding cassette (ABC) transporter family proteins [11,12]. Decreases in the expression of these receptor genes reduce toxin-receptor interactions and thus promote the evolution of Bt resistance in various insects [13,14]. Nevertheless, the mechanistic details of the transcriptional regulation of these midgut Cry receptor genes remain largely unknown.

The *ABCG1* gene (also known as *white*) was the first identified ABC transporter gene in arthropods [15] and participates in diverse physiological processes. In humans, the *ABCG1* gene plays important roles in cellular lipid homeostasis, cell proliferation, apoptosis, vasoconstriction, vasorelaxation, and several human diseases [16,17,18]. In insects, the *ABCG1* gene is responsible for color determination of the eye, serosa, or epidermis; behavior; and detoxification of toxic substances [19]. In crustaceans, the *ABCG1* gene is critical for the responses to acidic and alkaline conditions and for xenobiotic detoxification [20,21,22]. Our recent studies have suggested that *ABCG1* can also act as a functional midgut receptor of the Bt Cry1Ac toxin, and its reduced expression is closely linked to Bt Cry1Ac resistance [14,23]. Although the transcriptional regulation of *ABCG1* expression in humans has been explored in depth [18], information about this process in insects is sparse.

Intraspecific and interspecific variation in gene expression is common and is considered to favor adaptive evolution and species diversification [24]. Approaches used to explore the evolution of gene expression commonly focus on two components: *cis*-acting elements and *trans*-acting factors [24]. *cis*-acting mutations, including base mutations and insertions/deletions (indels), and changes in *trans*-acting factors are involved in the regulation of P450 genes that confer resistance to chemical insecticides in various insects [25,26,27,28,29,30,31,32,33]. The mitogen-activated protein kinase (MAPK) signaling pathway *trans*-regulates the differential expression of multiple midgut Cry1Ac receptor genes and non-receptor paralogous genes to mediate high-level resistance to the Bt Cry1Ac toxin in *Plutella xylostella* [14,23,34,35,36], suggesting that some MAPK-responsive transcription factors (TFs) are responsible for regulation of such downstream midgut genes, including *PxABCG1*. Nonetheless, the *cis*- and *trans*-factors that modulate the downregulation of midgut receptor genes, including *ABCG1,* in Bt-resistant insects are still unclear.

Here, we investigated the transcriptional regulation of the differential expression of the *PxABCG1* gene in *P. xylostella*. Our work shows that a Hox family TF, Antennapedia (Antp), interacts with its binding site (a *cis*-regulatory element, CRE) in the *PxABCG1* promoter of a susceptible strain to activate its expression. However, a *cis*-acting mutation makes Antp unable to bind to the CRE and regulate the *PxABCG1* gene, which leads to downregulation of *PxABCG1* gene expression and enhances resistance to the Cry1Ac toxin in *P. xylostella*. Our work on *cis*- and *trans*-regulation of the *PxABCG1* gene adds to the body of knowledge of the roles of *cis*- and *trans*-regulatory variations in environmental adaptation and contributes to a more complete understanding of the Bt resistance mechanism.

## 2. Results

### 2.1. Cloning and Analysis of the PxABCG1 Promoter in Bt-Susceptible and Bt-Resistant Strains

**Figure 1:**
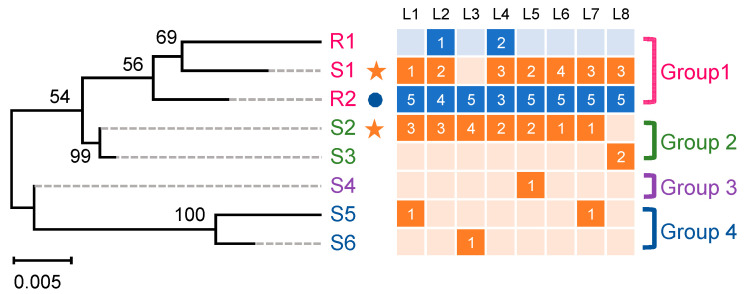
Phylogenetic relationships of *PxABCG1* promoter sequences amplified from the Bt-susceptible strain DBM1Ac-S and the Bt-resistant strain NIL-R. The phylogenetic tree was generated using the maximum likelihood (ML) method based on the model optimized using the Bayes Information Criterion with “complete deletion” as the gaps/missing data treatment and 1000 bootstrap replications. The classification of different *PxABCG1* promoter sequences is shown on the right. Promoter sequences of the *PxABCG1* gene were amplified from 8 susceptible DBM1Ac-S larvae and 8 resistant NIL-R larvae, respectively. L1 to L8 above represents each susceptible or resistant larva, and five clones from each larva were sequenced. Squares shaded in orange and blue represent susceptible and resistant larvae, respectively, and the number within the squares shows the number of the corresponding promoter detected in the 5 sequenced clones per larva. The orange star and the blue circle denote the major type of *PxABCG1* promoter sequences in the susceptible and resistant larvae, respectively.

A total of eight 5′-untranslated region (5′-UTR) sequences of the *PxABCG1* gene containing abundant single-nucleotide polymorphisms (SNPs) and fragment indels were obtained from eight individuals, each of the Bt-susceptible DBM1Ac-S and Bt-resistant NIL-R strains, respectively (Figure S1). Notably, all larvae of the resistant strain exhibited only two 5′-UTR sequences (R1 and R2), while larvae of the susceptible strain obtained six corresponding sequences (S1–S6) (Figure S1). A phylogenetic analysis was performed to clarify the evolutionary relationships among these different 5′-UTR sequences of the *PxABCG1* gene. The sequences clustered into four different groups (designated groups 1 to 4). The two sequences of the resistant strain were most similar to S1 of the susceptible strain; these three sequences were clustered into group 1. The other three groups were composed of different sequences of the susceptible strain (Figure 1). All the 5′-UTR sequences of the *PxABCG1* gene shared high sequence identity ranging from 94.47% to 100% between and within groups (Figure S2). R2 was the dominant 5′-UTR sequence for the resistant strain, which was amplified in all eight individuals (Figure 1). Of the six 5′-UTR sequences in the susceptible strain, S1 and S2 were detected in six of the eight individuals, while the other sequences (S3–S6) were detected only in very few larvae (Figure 1). Therefore, the S1 and S2 sequences were the main promoters for the susceptible strain (Figure 1).

### 2.2. PxABCG1 Promoter Activity Differs between Bt-Susceptible and Bt-Resistant Strains

**Figure 2:**
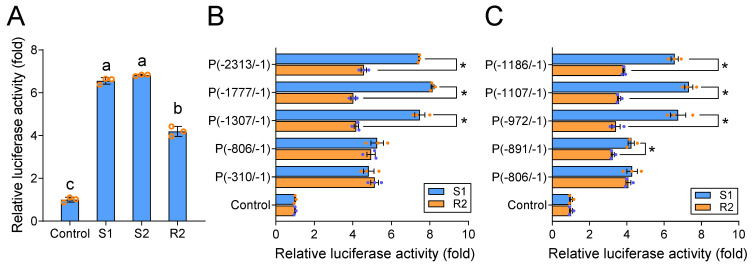
Detection of promoter activity by dual luciferase reporter assay. (**A**) Analysis of the activity of the primary *PxABCG1* resistance promoter (R2) and the main *PxABCG1* susceptibility promoter (wild-type S1/S2). The relative luciferase activity (firefly luciferase activity/*Renilla* luciferase activity) of different pGL4.10-promoter plasmids was normalized to that of the control pGL4.10 vector. The values shown are the means and the corresponding standard error of the mean (SEM) values for three biological replicates and four technical replicates. The significance of differences was determined by one-way ANOVA with Duncan’s test (*p* < 0.05). Different letters on the bars indicate significant differences. (**B**) Activity of progressive 5′ deleted recombinants of the S1 and R2 promoters after shortening from −2313 to −310. Student’s t-test was used for statistical analysis (*, *p* < 0.05). (**C**) Activity of progressive 5′ deletion constructs created through shortening of the sequence from −1186 to −806. Student’s t-test was used for statistical analysis (*, *p* < 0.05).

To explore whether the differences in the 5′-UTR sequence of the *PxABCG1* gene between the Bt-susceptible and Bt-resistant strains can affect promoter activity and thus lead to differential expression of the *PxABCG1* gene, the full-length sequences of R2, S1, and S2 were subcloned into the pGL4.10 plasmid. The resulting plasmids were transfected into S2 cells, and the promoter activity was detected. The results showed that the activity of S1 and S2 was significantly higher than that of R2 (Figure 2A), indicating that the different 5′-UTRs result in different promoter activity levels and affect the transcript levels of the *PxABCG1* gene in Bt-susceptible and Bt-resistant strains.

To further locate the sites leading to the different activity levels of the susceptible and resistant promoters, we used S1 and R2 as templates to construct a series of progressive 5′ deleted recombinants and measured their activity levels. When the promoters were truncated from −1307 to −806, the promoter activity of S1 was significantly decreased and became similar to the R2 activity (Figure 2B), suggesting that the elements responsible for the differential activity of the *PxABCG1* promoter S1 are located within the fragment from −1307 to −806 (Figure 2B).

To narrow down the location of the possible *cis*-acting element, we further shortened the S1 and R2 promoter regions from −1307 to −806. The differential activity between S1 and R2 remarkably reduced when the promoters were truncated from −972 to −891, and disappeared once sequences upstream of position −806 were removed (Figure 2C). These results suggest that the promoter region with differential activity between susceptible and resistant strains is located in the region from −972 to −806, especially the region between −972 and −891.

### 2.3. A cis-Acting Mutation in the Binding Site of Antp Reduces PxABCG1 Promoter Activity

**Figure 3:**
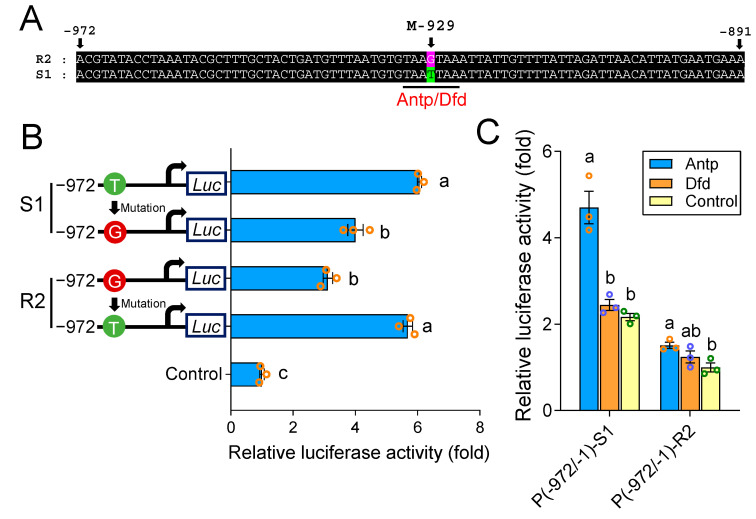
Analysis of the point mutation between −972 and −891. (**A**) Nucleotide sequence alignment of the fragment from −972 and −891 in *PxABCG1* promoters S1 and R2. (**B**) Activity of the promoters P(-972/-1)-S1 and P(-972/-1)-R2 with site-directed mutagenesis at M-929. The empty pGL4.10 vector was used as a control. The significance of differences was determined by one-way ANOVA with Duncan’s test (*p* < 0.05). Different letters on the bars indicate significant differences. (**C**) Effects of Antp and Dfd on the activity levels of *PxABCG1* promoters S1 and R2. The empty pAc5.1 vector was used for controls. The significance of differences was determined by one-way ANOVA with Duncan’s test (*p* < 0.05). Different letters on the bars indicate significant differences.

Nucleotide sequence alignment of the region from −972 to −891 showed that there was only a single point mutation (M-929) difference at nucleotide −929 between the S1 and R2 promoters (Figure 3A). To probe whether this mutation can result in a difference in promoter activity, we used the P(-972/-1) constructs of S1 and R2 as templates and performed directed mutation at this site. The point mutation at M-929 (from T to G) significantly reduced the activity of P(-972/-1)-S1, whereas the change from G to T elevated the activity of P(-972/-1)-R2 (Figure 3B). This indicated that M-929 was primarily responsible for the difference in *PxABCG1* promoter activity between −972 and −891. A binding site (CRE, TAATTAA, −932 to −925) of Antp and Deformed (Dfd) was predicted to occur near M-929 in the S1 promoter, and the point mutation in M-929 led to CRE disappearance in the R2 promoter of the resistant strain (Figure 3A), suggesting that the disappearance of the CRE may cause Antp and/or Dfd to be unable to bind to the promoter and thus reduces *PxABCG1* expression in the Bt-resistant strain. To test this hypothesis, expression vectors for Antp and Dfd were constructed and cotransfected with P(-972/-1). The data showed that Dfd did not obviously affect S1 and R2 but that Antp enhanced the activity of S1 but not that of R2 (Figure 3C), indicating that Antp is involved in *PxABCG1* expression regulation in the susceptible strain.

### 2.4. A cis-Acting Mutation Causes Antp to Fail to Bind to the Promoter and Regulate PxABCG1

**Figure 4:**
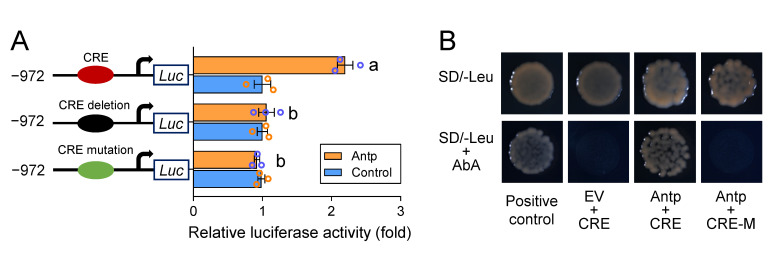
Antp positively regulates *PxABCG1* promoter activity through the CRE. (**A**) Effect of Antp on the activity of the *PxABCG1* promoter with either a deletion or a mutation in the CRE. The CRE (TAATTAA, −932 to −925) in P(-972/-1)-S1 was deleted or mutated to TAAGTAA. Antp was cotransfected with P(-972/-1)-S1 containing a normal CRE (red ellipse), lacking a CRE (black ellipse), or containing a mutant CRE (green ellipse). The empty pAc5.1 vector was used as a control. One-way ANOVA followed by Duncan’s test was used for statistical analysis (*p* < 0.05). Different letters on the bars indicate significant differences. (**B**) Investigation of the direct interaction between Antp and the CRE using a Y1H assay. Bait vectors containing three tandem repeats with the normal or mutant CRE and a prey vector containing Antp were transferred into the Y1HGold yeast strain. The yeast was grown on SD/-Leu selective medium with or without AbA. EV, empty prey vector; positive control, pGADT7-p53 + pABAi-p53.

To further verify whether the *cis*-acting mutation in the predicted binding site affects the positive regulation of Antp in the *PxABCG1*-susceptible promoter, the CRE (TAATTAA, −932 to −925) in P(-972/-1)-S1 was deleted or mutated to TAAGTAA. Antp was then cotransfected into S2 cells with P(-972/-1)-S1 containing a normal, deleted, or mutant CRE. Antp could not trigger the activity of the *PxABCG1* promoter after deletion or mutation of the CRE (Figure 4A). A yeast one-hybrid (Y1H) assay was further performed to detect the interaction between Antp and the normal or mutant CRE. Y1HGold strains transformed with Antp, and the normal CRE grew normally on the medium lacking leucine (Leu) and supplemented with aureobasidin A (AbA) (Figure 4B). These results indicate that Antp positively regulates the expression of the *PxABCG1* gene through the CRE in the promoter of the susceptible strain and that the *cis*-acting mutation in the Antp CRE prevents the binding and regulation of Antp.

### 2.5. Antp-Mediated Positive Regulation of PxABCG1 In Vivo Is Eliminated in the Bt-Resistant Strain

The TF Antp, which belongs to the Hox family, contains a conserved homeodomain in the C-terminus responsible for specific DNA binding and a YPWM motif adjacent to the homeobox in the N-terminus that typically mediates protein dimerization (Figures S3 and S4) [37]. A phylogenetic analysis of Antp was performed to clarify the evolutionary history of Antp proteins in diverse insects. The Antp proteins were evolutionarily conserved and clearly clustered into groups corresponding to the insect orders (Figure S3).

**Figure 5:**
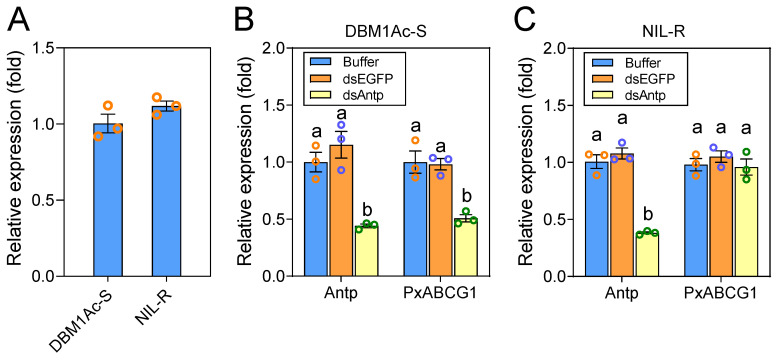
Effect of Antp on *PxABCG1* expression in vivo. (**A**) Relative expression of the midgut *Antp* gene in 4th-instar larvae of the susceptible strain DBM1Ac-S and the resistant strain NIL-R. The *RPL32* gene was used as an internal control. The average relative expression level and standard error of the mean (SEM) of three independent replicates are presented. Student’s t-test was used for statistical analysis (*p* < 0.05). (**B**) Relative expression of *Antp* and *PxABCG1* in larvae of the susceptible strain DBM1Ac-S at 48 h post injection with buffer, dsEGFP, or dsAntp. The expression levels of *Antp* or *PxABCG1* in the control larvae injected with buffer were set as 1. Different letters among groups indicate statistically significant differences (*p* < 0.05; Duncan’s test; n = 3). Different letters on the bars indicate significant differences. (**C**) Relative expression of *Antp* and *PxABCG1* in larvae of the resistant strain NIL-R at 48 h post injection with buffer, dsEGFP, or dsAntp. Different letters on the bars indicate significant differences.

To investigate whether Antp participates in resistance to Cry1Ac toxin in *P. xylostella* by controlling the Bt receptor gene *PxABCG1*, real-time quantitative PCR (qPCR) was first conducted to detect *Antp* expression levels in the midgut tissues of the Bt-susceptible and Bt-resistant strains. The transcript level of the midgut *Antp* gene was not significantly different between the susceptible strain DBM1Ac-S and the resistant strain NIL-R (Figure 5A), excluding the possibility that variation in *Antp* expression is related to Bt resistance. Furthermore, RNA interference (RNAi) was performed in susceptible and resistant larvae to investigate whether Antp regulates *PxABCG1* expression in vivo. After *Antp* silencing, the expression level of the *PxABCG1* gene was decreased significantly in the susceptible strain DBM1Ac-S (Figure 5B). However, downregulation of Antp did not have an effect on *PxABCG1* expression in the resistant strain NIL-R (Figure 5C). These in vivo results indicate that Antp positively regulates *PxABCG1* gene expression in the susceptible strain but is unable to regulate *PxABCG1* in the Bt-resistant strain.

## 3. Discussion

Variations in gene expression, which are ubiquitous within populations and between species, are considered to be the raw material for evolution and to contribute to adaptive evolution [24,38,39]. Decreased expression of midgut Bt receptor genes is one of the primary reasons for insects developing high-level Bt resistance. However, little is known about the transcriptional regulation of the differential expression of these genes. In this study, we revealed that Antp positively regulates the expression of the Cry1Ac receptor gene *PxABCG1* and that a *cis*-acting mutation causes Antp to fail to activate *PxABCG1* expression, thus increasing resistance to the Cry1Ac toxin in *P. xylostella*.

Numerous studies support the idea that *cis*-variation of individual or multiple genes contributes to the evolution of gene expression and to environmental adaptability in eukaryotes [40,41]. It is generally believed that *cis*-evolution is less pleiotropic and more common in “structural” genes such as protease genes, which do not directly affect the expression of other genes compared with regulatory genes [24]. In insects, *cis*-variation, including base mutation and fragment insertion/deletion in the 5′-UTR, can cause constitutive overexpression of P450 genes and result in phenotypes of resistance to chemical pesticides [25,26,27,28,29,30,31,32]. For example, transposable element insertions into the 5′-UTR of the *Cyp6g1* gene cause overexpression of this gene at the transcriptional level, conferring DDT resistance in *Drosophila melanogaster* [25,26]. In addition, a single nucleotide change in a core promoter is involved in *CYP9M10* gene overexpression in pyrethroid-resistant *Culex quinquefasciatus* [27]. Overexpression of *CYP6CY3*, which confers resistance to the plant alkaloid nicotine and neonicotinoids in *Myzus persicae*, is related to the insertion of dinucleotide microsatellites in the promoter [28]. Multiple mutations in *cis*-acting elements lead to increased expression of *CYP6FU1* and confer resistance to deltamethrin in *Laodelphax striatellus* [29]. Moreover, *cis*-regulatory variants of *CYP6P9a*- and *CYP6P9b*-mediated gene overexpression are associated with pyrethroid resistance in the African malaria vector *Anopheles funestus* [30,31]. Recently, a *cis*-acting mutation was found to increase *CYP321A8* expression and chlorpyrifos resistance in *Spodoptera exigua* [32]. Rather than P450 gene overexpression, which enhances metabolic detoxification and causes insect resistance to chemical insecticides, the molecular mechanism of insect resistance to Bt toxins is closely linked to the downregulation of midgut Bt receptor genes. Our study provides evidence of a *cis*-acting mutation that reduces the expression of the midgut Bt receptor gene *PxABCG1*. This result advances understanding of how *cis*-acting mutations contribute to the subtle control of gene expression and the evolution of Bt resistance.

**Figure 6:**
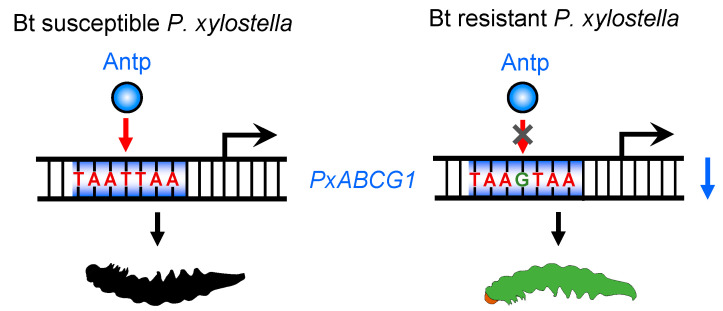
Schematic of the transcriptional regulation of reduced *PxABCG1* expression. In the Cry1Ac resistant strain *P. xylostella*, a *cis*-acting mutation in the binding site of Antp prevents Antp binding and downregulates *PxABCG1* expression, thereby enhancing larval resistance to Cry1Ac toxin. The black larva denotes larval death, and the green larva represents larval survival.

Functional divergence of *cis*-acting elements resulting from nucleotide substitutions, insertions, and/or deletions usually disrupts TF binding [42]. Some potential binding sites of TFs were thought to be changed due to *cis*-acting mutations in P450 gene promoters in the studies producing the abovementioned findings; however, corresponding specific *trans*-factors that act in concert with predicted binding sites have rarely been identified and verified. A recent study demonstrated that a *cis*-acting mutation increases *CYP321A8* expression by creating a binding site for Knirps [32]. In this study, we demonstrated that the binding site of Antp was altered from the *PxABCG1* promoter in the resistant strain and that the *cis*-acting mutation disrupted Antp binding and decreased *PxABCG1* expression, conferring resistance to the Cry1Ac toxin in *P. xylostella* (Figure 6). Increased understanding of the interaction between *cis*-acting elements and *trans*-acting factors related to Bt resistance will enable more complete and accurate appreciation of the Bt resistance mechanism. Notably, the *cis*-variation in the *PxABCG1* promoter can be exploited in a DNA-based assay to detect the frequency and distribution of midgut Bt receptor-mediated Cry1Ac resistance in *P. xylostella* [30,31], which will make a crucial contribution to Bt resistance management.

Hox family members, which form a highly conserved subclass of the homeodomain superfamily, are master regulators that determine cellular fates during embryonic morphogenesis and maintain tissue architecture [43,44]. Antp, a Hox family protein, was originally discovered in *D. melanogaster*, and its mutation causes abnormal body formation during embryogenesis [45]. Since the discovery of Antp, studies in *Bombyx mori* have demonstrated that Antp regulates the region-specific expression of multiple silk protein genes, including *sericin-1*, *sericin-3*, *fhxh4*, and *fhxh5*, in the middle silk gland [46,47]. Although the important regulatory role of Antp in normal growth and development has been corroborated, no study has investigated its function in insect resistance to chemical pesticides or biological pesticides. Here, we verified a new target gene of Antp, the Cry1Ac receptor gene *PxABCG1*, which is activated by Antp via a classical binding site. A *cis*-acting mutation in the *PxABCG1* promoter results in the failure of Antp to regulate *PxABCG1*, thus enhancing larval resistance to the Cry1Ac toxin. Conserved TFs, including Hox proteins, can acquire new target genes through evolutionary processes [48]. For example, alterations in some target genes of Ultrabithorax (Ubx) are associated with the diversification of insect wings [49,50]. Considering that the other roles of Antp remain largely unexplored, identification of more target genes and functional binding sites of Antp will contribute to our understanding of the in vivo functions of Hox proteins.

Despite the finding that a *cis*-acting mutation is related to reduced expression of the *PxABCG1* gene, in vivo evidence is needed to verify the effects of this point mutation on *PxABCG1* expression and Cry1Ac resistance in *P. xylostella* further. Recently, the novel CRISPR/Cas9 genome editing technique has been applied to probe gene functions in diverse species [51]. This powerful tool also plays a critical role in the dissection of the transcriptional regulation of functional genes by enabling the generation of *cis*-acting mutations or mutagenesis of the coding DNA sequences of *trans*-acting factors [37]. However, the mutation of *trans*-acting factors usually leads to complete loss of function with pleiotropic effects [52], especially for some regulators essential for normal growth and development, such as Hox factors, and dysregulation of these genes can lead to abnormal development and malignant tumors in humans [44]. In contrast, editing of the *cis*-acting elements of target genes provides the possibility of generating a series of alleles with different transcript levels, thus assisting in the exploration of the effect of *cis*-variation on gene expression and the fine-tuning of target expression [37,52]. In the tomato, new alleles with varying expression levels have been generated to optimize the inflorescence architecture by using CRISPR to target the *cis*-acting elements of the *SEPALLATA4* and *FRUITFULL* genes [53]. CRISPR/Cas9-targeted mutagenesis of the Vrille (Vri) binding site in the enhancer in the male *Daphnia magna* genome results in reduced expression of the *Dsx1* gene [54]. We have also successfully applied the CRISPR/Cas9 system to knock out the *PxAPN1*, *PxAPN3a*, *PxABCC2*, and *PxABCC3* genes in *P. xylostella* in order to validate the roles of these genes in Cry1Ac resistance [23,36]. Functional verification of *cis*-acting mutations with CRISPR/Cas9 technology will be conducive to illuminating the in vivo effect of *cis*-variation on *PxABCG1* expression in *P. xylostella*.

Our previous studies have demonstrated that the activated MAPK signaling pathway *trans*-regulates the differential expression of multiple midgut Bt receptor genes, including *PxABCG1,* to confer high-level resistance to the Cry1Ac toxin in *P. xylostella* [14,23,34,35,36], indicating that one or more TFs downstream respond to the MAPK signaling pathway to modulate the expression of these genes. Indeed, our recent study has revealed that MAPK-activated PxJun represses the expression of the midgut Bt receptor gene *PxABCB1* and thus increases larval resistance to the Cry1Ac toxin [55]. Thus, in addition to *cis*-variation, *trans*-acting factors downstream of MAPK likely also participate in the downregulation of the *PxABCG1* gene. This possibility will be investigated in future studies.

## 4. Materials and Methods

### 4.1. Insect Strains and Cell Line

The Bt-susceptible *P. xylostella* strain DBM1Ac-S and the near-isogenic Cry1Ac-resistant strain NIL-R were used in this study, as described in detail previously [35,56,57]. Briefly, the DBM1Ac-S strain has been kept continuously for more than 10 years in our laboratory without exposure to any pesticides. The NIL-R strain exhibits over 4000-fold greater resistance to the Bt Cry1Ac protoxin than the susceptible DBM1Ac-S strain. The larvae were reared on Jing Feng No. 1 cabbage (*Brassica oleracea* var. *capitata*) at 25 °C under 65% relative humidity (RH) and a 16:8 (light:dark) photoperiod. The adults were supplied with a 10% honey/water solution.

*Drosophila* S2 cells for the dual-luciferase reporter assay were cultured in a HyClone SFX-insect medium (HyClone, Logan, UT, USA) supplemented with penicillin-streptomycin (Gibco, Rockville, MD, USA) at 27 °C.

### 4.2. Toxin Preparation and Bioassay

The Cry1Ac protoxin preparation and leaf-dip bioassay were carried out as described previously [14,58]. Briefly, the Cry1Ac protoxin was extracted and purified from Bt var. *kurstaki* (Btk) strain HD-73 and then quantified and stored in 50 mM Na_2_CO_3_ (pH 9.6) at −20 °C for subsequent use. A three-day leaf-dip bioassay was performed using third-instar larvae with ten per group and four replicates. The control mortality did not exceed 5%.

### 4.3. Extraction of DNA and RNA

Genomic DNA (gDNA) was extracted from individual fourth-instar larvae from the Bt-susceptible strain DBM1Ac-S and the resistant strain NIL-R using a TIANamp Genomic DNA Kit (Tiangen, Beijing, China). RNA was isolated using TRIzol reagent (Invitrogen, Carlsbad, CA, USA) from midgut tissue that was dissected from fourth-instar larvae in ice-cold insect Ringer’s solution (130 mM NaCl, 0.5 mM KCl, 0.1 mM CaCl_2_). First-strand cDNA used for gene cloning or qPCR was synthesized using a PrimeScript II First Strand cDNA Synthesis Kit (Takara, Dalian, China) or a PrimeScript RT Kit (with gDNA Eraser, Perfect Real Time) (Takara, Dalian, China) according to the manufacturers’ protocols. The obtained samples were stored at −20 °C until use.

### 4.4. Cloning of the Promoter and TFs

A pair of specific PCR primers (Table S1) was designed to amplify the *PxABCG1* promoter based on the 5′-UTR sequence of the *PxABCG1* gene in the *P. xylostella* genome from the DBM-DB (http://116.62.11.144/DBM/index.php, accessed date: 4 November 2019) and LepBase (http://ensembl.lepbase.org/Plutella_xylostella_pacbiov1/Info/Index, accessed date: 4 November 2019). Then, large-scale cloning and sequencing of the *PxABCG1* promoter were conducted for the Bt-susceptible DBM1Ac-S strain and the resistant NIL-R strain (gDNA as template, 8 samples per strain, and 5 positive clones of each sample for sequencing) to identify potential sequence variations. PrimeSTAR Max DNA Polymerase (Takara, Dalian, China) was used for PCR amplification following the manufacturer’s protocol. The PCR products were subsequently purified and subcloned into *pEASY*-T1 vectors (TransGen, Beijing, China) for further sequencing.

The coding sequences (CDSs) of *Antp* and *Dfd* in *P. xylostella* were retrieved from the GenBank database (https://www.ncbi.nlm.nih.gov/, accessed date: 7 June 2020) under accession numbers XM_011557407 and XM_038120187, respectively. The CDSs were further corrected with our previous *P. xylostella* midgut transcriptome database [59]. The full-length CDSs were amplified using their corresponding specific primers (Table S2).

### 4.5. Bioinformatic Analysis

The obtained CDSs of the TFs were translated into amino acid sequences using the ExPASy translate tool (https://web.expasy.org/translate/, accessed date: 19 June 2020). The DNA and protein sequences were analyzed and aligned using DNAMAN 7.0 software (Lynnon Biosoft, USA). Multiple sequence alignment was conducted using Clustal Omega (http://www.ebi.ac.uk/Tools/msa/clustalo/, accessed date: 13 March 2021), and the results were further formatted using GeneDoc 2.7 software (http://genedoc.software.informer.com/2.7/, 13 March 2021). Potential CREs for TF binding in the *PxABCG1* promoter were predicted with the JASPAR database (http://jaspar.genereg.net, accessed date: 6 June 2020) and the PROMO virtual laboratory (http://alggen.lsi.upc.es/cgi-bin/promo_v3/promo/promoinit.cgi?dirDB=TF_8.3, accessed date: 6 June 2020). The sequence similarity among different promoter sequences was analyzed using the BLAST at the NCBI website (https://blast.ncbi.nlm.nih.gov, 16 December 2019). A phylogenetic tree to clarify the phylogenetic relationships of different 5′-UTR sequences was generated using the maximum likelihood (ML) method with 1000 bootstrap replicates in MEGA 7.0 software (https://www.megasoftware.net/, 16 December 2019). The conserved domains of the Antp protein were analyzed using the Conserved Domain Database (CDD) at NCBI (https://www.ncbi.nlm.nih.gov/cdd/, 14 March 2021). A phylogenetic tree of Antp proteins in different insects was built using MEGA 7.0 software with the neighbor-joining (NJ) method.

### 4.6. Dual-Luciferase Assay

The two most common sequences of *PxABCG1* promoters in the susceptible strain (S1 and S2) and the primary promoter in the resistant strain (R2) were used for analysis and for a comparison of promoter activity. The full-length promoter and a series of 5′-truncated promoters were amplified using specific primers (Table S1) and subcloned into the firefly luciferase reporter vector pGL4.10 (Promega, Madison, WI, USA) to prepare pGL4.10-promoter constructs. Promoter fragments with CRE deletion or point mutation were obtained by gene synthesis (TsingKe, Nanjing, China). The CDSs of the TFs were amplified using their corresponding primers (Table S2) and subcloned into the pAc5.1/V5-His B (hereinafter called “pAc5.1”) expression vector (Invitrogen, Carlsbad, CA, USA). The pGL4.73 vector (Promega, Madison, WI, USA) containing a *Renilla* luciferase gene was used as an internal control.

Cell transfection was carried out using Lipofectamine 2000 transfection reagent (Thermo Fisher Scientific, Waltham, MA, USA). S2 cells were cultured in 24-well plates at a density of 5 × 10^5^ cells/well. To detect promoter activity, the promoter constructs (600 ng) were cotransfected with the pGL4.73 plasmid (200 ng), and the empty pGL4.10 vector was used as a control. To evaluate the regulatory effects of TFs on the promoter, TF expression plasmids (600 ng), promoter constructs (200 ng), and the pGL4.73 vector (100 ng) were cotransfected into S2 cells, and the empty pAc5.1 plasmid was used as a control. After 48 h of transfection, the cells were collected, and the luciferase activity was measured on a GloMax 96 Microplate Luminometer (Promega, Madison, WI, USA) by using a Dual-Luciferase Reporter Assay System (Promega, Madison, WI, USA) according to the manufacturer’s protocol. The relative luciferase activity (firefly luciferase activity/*Renilla* luciferase activity) of different pGL4.10-promoter plasmids was normalized to that of the control pGL4.10 vector. Each experiment was replicated three times. One-way ANOVA followed by Duncan’s test was used for analysis of the significant differences (*p* < 0.05).

### 4.7. Y1H Assay

A Y1H assay was performed to verify the direct interaction between Antp and the normal/mutant CRE using a Matchmaker Gold Yeast One-Hybrid System (Clontech, Mountain View, CA, USA) according to the recommended protocol. Bait plasmids (CRE and CRE-M) were generated by ligating three tandem repeats that contain the normal CRE (5′- taatgtgTAATTAAattatt-3′) or mutant CRE (5′-taatgtgTAAGTAAattatt-3′) into the pAbAi vector between the *Xho*I and *Hind*III restriction sites. The bait plasmids were linearized with *Bst*BI and integrated into Y1HGold yeast to generate the bait strains and were then selected on SD/-Ura medium. The minimum concentration of AbA that inhibited the normal growth of the bait strains on SD/-Ura medium was 600 ng/mL. The prey plasmid containing Antp was constructed by subcloning the CDS into the pGADT7 vector, which was then transformed into the bait strains. Selection was performed on a SD/-Leu medium with 600 ng/mL AbA. The positive control was the Y1HGold strain cotransformed with the pGADT7-p53 and pAbAi-p53 plasmids, and the negative control was the Y1HGold strain cotransformed with the empty vector pGADT7 and the normal pAbAi-CRE plasmid.

### 4.8. qPCR Analysis

As mentioned previously [14,34], the expression of the *Antp* and *PxABCG1* genes was quantified by qPCR using the specific primers listed in Table S3. The qPCR experiment was run on a QuantStudio 3 Real-Time PCR System (Applied Biosystems, USA) using FastFire qPCR PreMix (SYBR Green) (Tiangen, Beijing, China) according to the manufacturer’s instructions. Each experiment was performed with three biological replicates and four technical replicates. The relative expression levels were calculated using the 2^−ΔΔCT^ method and normalized to the level of the internal control ribosomal protein *L32* (*RPL32*) gene (GenBank accession no. AB180441). One-way ANOVA followed by Duncan’s test was used for analysis of the significant differences (*p* < 0.05).

### 4.9. RNAi

Silencing of *Antp* expression was carried out in larvae of the Bt-susceptible strain DBM1Ac-S and the resistant strain NIL-R via RNAi. Double-stranded RNA (dsRNA) preparation, dsRNA microinjection, midgut RNA extraction, qPCR, and bioassays were performed as mentioned previously [60]. Briefly, the cDNA fragments of *Antp* or *EGFP* to be used for dsRNA synthesis were amplified using gene-specific dsRNA primers containing a T7 promoter on the 5′ end (Table S3). Then, dsAntp and dsEGFP were synthesized using a T7 RiboMAX Express RNAi System (Promega, Madison, WI, USA). Thirty larvae were microinjected with buffer, dsEGFP (300 ng), or dsAntp (300 ng). Each treatment was performed with three biological replicates. The expression levels of *Antp* and *PxABCG1* were detected by qPCR. One-way ANOVA followed by Duncan’s test was used for analysis of the significant differences (*p* < 0.05).

## Data Availability

Not applicable.

## Supplementary Materials

The following are available online at https://www.mdpi.com/article/10.3390/ijms22116106/s1.
