# Supplementary material for: A cis-Acting Mutation in the PxABCG1 Promoter Is Associated with Cry1Ac Resistance in Plutella xylostella (L.)
Source: Int J Mol Sci. 2021 Jun 5;22(11):6106. doi: 10.3390/ijms22116106 (PMC8201282; doi:10.3390/ijms22116106)
Supplement: Supplementary file 1 [file ijms-22-06106-s001.zip › ijms-1224940-supplementary.pdf]

# Supplementary Information

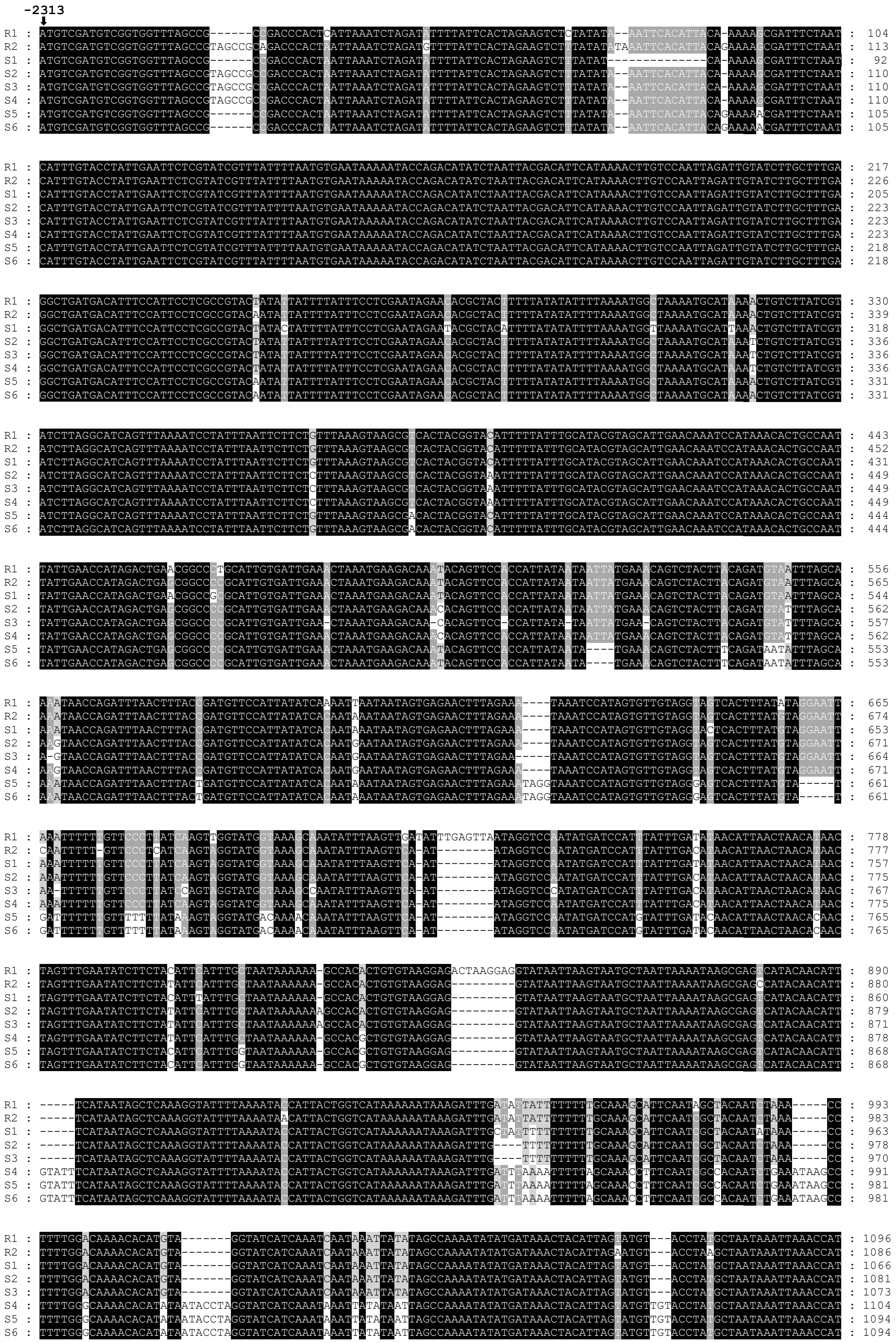

```
R1 : TAATTTAGGTTACTACGGTTATAAGGACT--ATTAAATTCATGAAAAGCTTCCTATGTTTGTGCTAAATAACTAATATTACTTCTAGTTGTTAACCATCGTTAAAAAAGATTA : 1207
R2 : TAATTTAGGTTACTACGGTTATAAGGACT--ATTAAATTCATGAAAAGCTTCCTATGTTTGTGCTAAATAACTAATATTACTTCTAGTTGTTAACCATCGTTAAAAAAGATTA : 1197
S1 : TAATTTAGGTTAATACTGTTATAAGGACT--ATTAAATTCACGAAAAGCTTCCTATGTTTGTGCTAAATAACTAATATTACTTCTAGTTGTTAACCATCGTTAAAAAAGATTA : 1177
S2 : TAATTTAGGTTACTACGGTTATAAGGACT--ATTAAATTCATGAAAAGCTTCCTATGTTTGTGCTAAATAACTAATATTACTTCTAGTTGTTAACCACCGTTAAAAA-GATTA : 1191
S3 : TAATTTAGGTTACTACGGTTATAAGGACT--ATTAAATTCATGAAAAGCTTCCTATGTTTGTGCTAAATAACTAATATTACTTCTAGTTGTTAACCACCGTTAAAAA-GATTA : 1183
S4 : TAATTTAGGTTACTACGGTTATAAGGACTCTATTAAATTCATGAAAAGCTTCCTATGTTTGTGCTAAATAACTAATATTACTTCTAGTTGTTAACCATCGTTAAAAAAGATTA : 1217
S5 : TAATTTAGGTTACTACGGTTATAAGGACTCTATTAAATTCATGAAAAGCTTCCTATGTTTGTGCTAAATAACTAATATTACTTCTAGTTGTTAACCATCGTTAAAAAAGATTA : 1207
S6 : TAATTTAGGTTACTACGGTTATAAGGACTCTATTAAATTCATGAAAAGCTTCCTATGTTTGTGCTAAATAACTAATATTACTTCTAGTTGTTAACCATCGTTAAAAAAGATTA : 1207

R1 : TCTTTTTGTGCAATGTAAATAGATACCATTATCGTATTTAATATTATACTTTCTTACCTAAACGA-TAAAATAAAATAAAAAATATGTTCATGGGAATGGTCGAGGTTAATTC : 1319
R2 : TCTTTTTGTGCAATGTAAATAGATACCATTATCGTATTTAATATTATACATTCTTACCTAAACGAATAAAATAAAATAAAAAATATGTTCATGGGAATGGTCGAGGTTAATTC : 1310
S1 : TCTTTTTGTGCAATGTAAATAGATACCATTATCGTATTTAATATTATACTTTCTTACCTAAACGAATAAAATAAAATAAAAAATATGTTCATGGGAATGGTCGAGGTTAATTC : 1290
S2 : TCTTTTTGTGCAATGTAAATAGATACCATTATCGTATTTAATATTATACTTTCTTACCTAAACGAATAAAATAAAATAAAAAATATGTTCATGGGAATGGTCGAGGTTAATTC : 1304
S3 : TCTTTTTGTGCAATGTAAATAGATACCATTATCGTATTTAATATTATACTTTCTTACCTAAACGAATAAAATAAAATAAAAAATATGTTCATGGGAATGGTCGAGGTTAATTC : 1296
S4 : TCTTTTTGTGCAATGTAAATAGATACCATTATCGTATTTAATATTATACTTTCTTACCTAAACGAATAAAATAAAATAAAAAATATGTTCATGGGAATGGTCGAGGTTAATTC : 1330
S5 : TCTTTTTGTGCAATGTAAATAGATACCATTATCGTATTTAATATTATACTTTCTTACCTAAACGAATAAAATAAAATAAAAAATATGTTCATGGGAATGGTCGAGGTTAATTC : 1320
S6 : TCTTTTTGTGCAATGTAAATAGATACCATTATCGTATTTAATATTATACTTTCTTACCTAAACGAATAAAATAAAATAAAAAATATGTTCATGGGAATGGTCGAGGTTAATTC : 1320

R1 : GCTTATGTAAGTAACCGATTGAATAAACGAAACGTATACCTAAATACGCTTTGCTACTGATGTTTAATGTGTAAGTAAATTATTGTTTTATTAGATTAACATTATGAATGAAA : 1432
R2 : GCTTATGTAAGTAACCGATTGAATAAACGAAACGTATACCTAAATACGCTTTGCTACTGATGTTTAATGTGTAAGTAAATTATTGTTTTATTAGATTAACATTATGAATGAAA : 1423
S1 : GCTTATGTAAGTAACCGATTGAATAAACGAAACGTATACCTAAATACGCTTTGCTACTGATGTTTAATGTGTAATTAAATTATTGTTTTATTAGATTAACATTATGAATGAAA : 1403
S2 : GCTTATGTAAGTAACCGATTGAATAAACGAAACGTATACCTAAATACGCTTTGCTACTGATGTTTAATGTGTAATTAAATTATTGTTTTATTAGATTAACATTATGAATGAAA : 1417
S3 : GCTTATGTAAGTAACCGATTGAATAAACGAAACGTATACCTAAATACGCTTTGCTACTGATGTTTAATGTGTAATTAAATTATTGTTTTATTAGATTAACATTATGAATGAAA : 1409
S4 : GCTTATGTAAGTAACCGATTGAATAAACGAAACGTATACCTAAATACGCTTTGCTACTGATGTTTAATGTGTAATTAAATTATTGTTTTATTAGATTAACATTATGAATGAAA : 1443
S5 : GCTTATGTAAGTAACCGATTGAATAAACGAAACGTATACCTAAATACGCTTTGCTACTGATGTTTAATGTGTAATTAAATTATTGTTTTATTAGATTAACATTATGAATGAAA : 1433
S6 : GCTTATGTAAGTAACCGATTGAATAAACGAAACGTATACCTAAATACGCTTTGCTACTGATGTTTAATGTGTAATTAAATTATTGTTTTATTAGATTAACATTATGAATGAAA : 1433

R1 : CGGTCTTAGTTAAAACGCTTCGATTGCGTGTAAGTACGTCGAACTACGGACGCTTGGTTAGGCGGCAATAACATAAAATAACTAAGAAAACATACTTAGTAAGTATACCTAGG : 1545
R2 : CGGTCTTAGTTAAAACGCTTCGATTGCGTGTAAGTACGTCGAACTATGGACGCTTGGTTAGGCGGCAATAACATAAAATAACTAAGAAAACATACTTAGTAAGTA--CCTAGG : 1534
S1 : CGGTCTTAGTTAAAACGCTTCGATTGCGTGTAAGTACGTCGAACTACGGACGCTTGGTTAGGCGGCAATAACATAAAATAACTAAGAAAACATACTTAGTAAGTA--CCTAGG : 1514
S2 : CGGTCTTAGTTAAAACGCTTCGATTGCGTGTAAGTACGTCGAACTATGGACGCTTGGTTAGGCGGCAATAACATAAAATAACTAAGAAAACATACTTAGTAAGTA--CCTAGG : 1528
S3 : CGGTCTTAGTTAAAACGCTTCGATTGCGTGTAAGTACGTCGAACTATGGACGCTTGGTTAGGCGGCAATAACATAAAATAACTAAGAAAACATACTTAGTAAGTA--CCTAGG : 1520
S4 : CGGTCTTAGTTAAAACGCTTCGATTGCGTGTAAGTACGTCGAACTACGGACGCTTGGTTAGGCGGCAATAACATAAAATAACTAAGAAAACATACTTAGTAAGTA--CCTAGG : 1554
S5 : CGGTCTTAGTTAAAACGCTTCGATTGCGTGTAAGTACGTCGAACTACGGACGCTTGGTTAGGCGGCAATAACATAAAATAACTAAGAAAACATACTTAGTAAGTA--CCTAGG : 1544
S6 : CGGTCTTAGTTAAAACGCTTCGATTGCGTGTAAGTACGTCGAACTACGGACGCTTGGTTAGGCGGCAATAACATAAAATAACTAAGAAAACATACTTAGTAAGTA--CCTAGG : 1544

R1 : AATAGGAACAGGTCTATAACGCCTGGCCGAATAGAAGATAATTCACCAAATAATAAATATAAATTAAATACGAATCAAGGTTTTTTGTATGTTATTCGCAATGTTATTGTCAA : 1658
R2 : AATAGGAACAGGTCTATAACGCCTGGCCGAATAGAAGATAATTCACCAAATAATAAATATAAATTAAATACGAATCAAGGTTTGTTGTATGTTATTCGCAATGTTATTGTCAA : 1647
S1 : AATAGGAACAGGTCTATAACGCCTGGCCGAATAGAAGATAATTCACCAAATAATAAATATAAATTAAATACGAATCAAGGTTTGTTGTATGTTATTCGCAATGTTATTGTCAA : 1627
S2 : AATAGGAACAGGTCTATAACGCCTGGCCGAATAGAAGATAATTCACCAAATAATAAATATAAATTAAATACGAATCAAGGTTTGTTGTATGTTATTCGCAATGTTATTGTCAA : 1641
S3 : AATAGGAACAGGTCTATAACGCCTGGCCGAATAGAAGATAATTCACCAAATAATAAATATAAATTAAATACGAATCAAGGTTTGTTGTATGTTATTCGCAATGTTATTGTCAA : 1633
S4 : AATAGGAACAGGTCTATAACGCCTGGCCCAATAGAAGATAATTCACCAAATAATAAATATAAATTAAATACGAATCAAGGTTTGTTGTATGTTATTCGCAATGTTATTGTCAA : 1667
S5 : AATAGGAACAGGTCTATAACGCCTGGCCGAATAGAAGATAATTCACCAAATAATAAATATAAATTAAATACGAATCAAGGTTTGTTGTATGTTATTCGCAATGTTATTGTCAA : 1657
S6 : AATAGGAACAGGTCTATAACGCCTGGCCCAATAGAAGATAATTCACCAAATAATAAATATAAATTAAATACGAATCAAGGTTTGTTGTATGTTATTCGCAATGTTATTGTCAA : 1657

R1 : ATAGAGCATAAGTTTACATTTATAATAGTTATGATAGCCATATTGCTAAATCATATTTAACTGTAGGGTTCATACATCATCTCAAGTCTGCATACAAATAGTTCCTTTGTCAT : 1771
R2 : ATAGAGCATAAGTTTACATTTATAATAGTTATGATAGCCATATTGCTAAATCATATTTAACTGTAGGGTTCATACATCATCTCAAGTCTGCATACAAATAGTTCCTTTGTCAT : 1760
S1 : ATAGAGCATAAGTTTACATTTATAATAGTTATGATAGCCATATTGCTAAATCATATTTAAATGTAGGGTTCATACATCATCTCAAGTCTGCATACAAATAGTTCCTTTGTCAT : 1740
S2 : ATAGAACATAAGTTTACATTTATAATAGTTATGATAGCCATATTGCTAAATCATATTTAACTGTAGGGTTCATACATCATCTCAAGTCTGCATACAAATAGTTCCTTTGTCAT : 1754
S3 : ATAGAACATAAGTTTACATTTATAATAGTTATGATAGCCATATTGCTAAATCATATTTAACTGTAGGGTTCATACATCATCTCAAGTCTGCATACAAATAGTTCCTTTGTCAT : 1746
S4 : ATAGAGCATAAGTTTACATTTATAATAGTTATGATAGCCATATTGCTAAATCATATTTAACTGTAGGGTTCATACATCATCTCAAGTCTGCATACAAATAGTTCCTTTGTCAT : 1780
S5 : ATAGAGCATAAGTTTACATTTATAATAGTTATGATAGCCATATTGCTAAATCATATTTAAATGTAGGGTTCATACATCATCTCAAGTCTGCATACAAATAGTTCCTTTGTCAT : 1770
S6 : ATAGAGCATAAGTTTACATTTATAATAGTTATGATAGCCATATTGCTAAATCATATTTAACTGTAGGGTTCATACATCATCTCAAGTCTGCATACAAATAGTTCCTTTGTCAT : 1770

R1 : GTGTCATTTTTAATCGTACTCGTATCGAAATGTAAAAGGTATTTCATTATAATTAAAATATTGTATAAATAAATAATTACCGAATAATTATTTGCTAAGCTCAAATATGTTTT : 1884
R2 : GTGTCATTTTTAATCGTACTCGTATCGAAATGTAAAAGGTATTTCATTATAATTAAAATATTGTATAAATAAATAATTACCGAATAATTATTTGCTAAGCTCAAATATGTTTT : 1873
S1 : GTGTCATTTTTAATCGTACTCGTATCGAAATGTAAAAGGTATTTCATTATAATTAAAATATTGTATAAATAAATAATTACCGAATAATTATTTGCTAAGCTCAAATATGTTTT : 1853
S2 : GTGTCATTTTTTATCGTACTCGTATCGAAATGTAAAAGGTATTTCATTATAATTAAAATATTGTATAAATAAATAATTACCGAATAATTATTTGCTAAGCTCAAATATGTTTT : 1867
S3 : GTGTCATTTTTTATCGTACTCGTATCGAAATGTAAAAGGTATTTCATTATAATTAAAATATTGTATAAATAAATAATTACCGAATAATTATTTGCTAAGCTCAAATATGTTTT : 1859
S4 : GTGTCATTTTTTATCGTACTCGTATCGAAATGTAAAAGGTATTTCATTATAATTAAAATATTGTATAAATAAATAATTACCGAATAATTATTTGCTAAGCTCAAATATGTTTT : 1893
S5 : GTGTCATTTTTAATCGTACTCGTATCGAAATGTAAAAGGTATTTCATTATAATTAAAATATTGTATAAATAAATAATTACCGAATAATTATTTGCTAAGCTCAAATATGTTTT : 1883
S6 : GTGTCATTTTTAATCGTACTCGTATCGAAATGTAAAAGGTATTTCATTATAATTAAAATATTGTATAAATAAATAATTACCGAATAATTATTTGCTAAGCTCAAATATGTTTT : 1883

R1 : TTTCATAAATAAACATTTACATACGATTATTTTTGTATTAATTTCTAATGTATAAAC-TCCTAAACATTATCAAACTTAACAATCTTTGTACTGTTATTTAACTCACAGGCAC : 1996
R2 : TTTCATAAATAAACATTTACATACGATTAGTTTTGTATTAATTTCTAATGTATAAAC-TCCTAAACATTATCAAACTTAACAATCTTCGTACTGTTA--AACTAACAGGCAC : 1983
S1 : TTTCATAAATAAACATTTACATACGATTATTTTTGTATTAATTTCTAATGTATAAACCTCCTAAACATTATCAAACTTAACAATCCTTGTACTGTTATTTAACTCACAGGCAC : 1966
S2 : TTTCATAAATAAACATTTACATACGATTACTTTTGTATTAATTTCCAATGTATAAAC-TCCTAAACATTATCAAACTTAACAATCTTCGTACTGTTA--AACTCACAGGCAC : 1977
S3 : TTTCATAAATAAACATTTACATACGATTACTTTTGTATTAATTTCCAATGTATAAAC-TCCTAAACATTATCAAACTTAACAATCTTCGTACTGTTA--AACTCACAGGCAC : 1969
S4 : TTTCATAAATAAACATTTACATACGATTACTTTTGTATTAATTTCCAATGTATAAAC-TCCTAAACATTATCAAACTTAACAATCTTCGTACTGTTA--AACTCACAGGCAC : 2003
S5 : TTTCATAAATAAACATTTACATACGATTATTTTTGTATTAATTTCTAATGTATAAACCTCCTAAACATTATCAAACTTAACAATCCTTGTACTGTTATTTAACTCACAGGCAC : 1996
S6 : TTTCATAAATAAACATTTACATACGATTAGTTTTGTATTAATTTCTAATGTATAAAC-TCCTAAACATTATCAAACTTAACAATCTTCGTACTGTTA--AAACTCACAGGCAC : 1993

R1 : GTCCTTTGTGT------TAATATTCGTCTCTTTATTGTAACAGCTTATCTCAATCCCGAACTTCAATAGACATGAAACTACATTTGGCGCTAAGCAAAGCCAAGCAGTCCTAG : 2103
R2 : GTCCTTTGTGTAGGTATTATTAATAACTGTCTCTTTGTAACAGCTTATCTCAATCCCGAACTTCAATAGACATGAAACCACATTTCGCGCTAAGCAAAGCCAAGCACTGCTAG : 2096
S1 : GTCCTTTGTGTA----TTATTAATAACTGTCTCTTTGTAACAGCTTATCTCAATCCCGAACTTCAATAGACATGAAACTACATTTCGCGCTAAGCAGAGCCAAGCTCTGCTAG : 2075
S2 : GTCCTTTGTGTA----TTAGTAATAACTGTCTCTTTGTAACAGCTTATCTCAATCCCGAACTTCAATAGACATGAAACTACATTTCGCGCTAAGCAGAGCAAAGCTCTGCTAG : 2086
S3 : GTCCTTTGTGTA----TTAGTAATAACTGTCTCTTTGTAACAGCTTATCTCAATCCCGAACTTCAATAGACATGAAACTACATTTCGCGCTAAGCAGAGCAAAGCTCTGCTAG : 2078
S4 : GTCCTTTGTGTA----TTAGTAATAACTGTCTCTTTGTAACAGCTTATCTCAATCCCGAACTTCAATAGACATGAAACTACATTTCGCGCTAAGCAGAGCAAAGCTCTGCTAG : 2112
S5 : GTCCTTTGTGTAT----TATTAATAACTGTCTCTTTGTAACAGCTTATCTCAATCCCGAACTTCAATAGACATGAAACTACATTTCGCGCTAAGCAGAGCCAAGCTCTGCTAG : 2105
S6 : GTCCTTTGTGT------TAATATTCGTCTCTTTATTGTAACAGCTTATCTCAATCCCGAACTTCAATAGACATGAAACTAAATT-CGCGCCAAGCAAAACCAAGCACTGCTAG : 2099

R1 : ATGAAACACCTCCCCAAAGTTACCAGTCCAGAGCTCATTAGAAGAGATACGGAGAGTAAGGCGCCGCTCGAGTCAGTAGGCTCATTTCGACACAACGTGCGCGCGCGGCCGAC : 2216
R2 : ATGAAACACCTCCCCAAAGTTACCAGTCCAGAGCTCATTAGAAGAGACACGAAGAGTAAGACGCCGCTCGAGTCAGTAGGCTCATTTCGACACAACGTGCGCGCGCGGCCGAC : 2209
S1 : ATGAAACACCTCCCCAAAGTTACCAGTCCAGAGCTCATTAGAAGAGACACGAAGAGTAAGGTGCCGCTCGAGTCAGTAGGCTCATTTCGACACAACGTGCGCGCGCGGCCGAC : 2188
S2 : ATGAAACACCTCCCCAAAGTTACCAGTCCAGAGCTCATTAGAAGAGATACGAAGAGTA-GGCGCCGCTCGAGTCAGTAGGCTCATTTCGACACAACGTGCGCGCGCGGCCGAC : 2198
S3 : ATGAAACACCTCCCCAAAGTTACCAGTCCAGAGCTCATTAGAAGAGATACGAAGAGTA-GGCGCCGCTCGAGTCAGTAGGCTCATTTCGACACAACGTGCGCGCGCGGCCGAC : 2190
S4 : ATGAAACACCTCCCCAAAGTTACCAGTCCAGAGCTCATTAGAAGAGATACGAAGAGTA-GGCGCCGCTCGAGTCAGTAGGCTCATTTCGACACAACGTGCGCGCGCGGCCGAC : 2224
S5 : ATGAAACACCTCCCCAAAGTTACCAGTCCAGAGCTCATTAGAAGAGACACGAAGAGTAAGGTGCCGCTCGAGTCAGTAGGCTCATTTCGACACAACGTGCGCGCGCGGCCGAC : 2218
S6 : ATGAAACACCTCCCCAAAGTTACCAGTCCAGAGCTCATTAGAAGAGATGTGAAGAGTAAGGCGTCGCTCGAGTCAGTAGGCTCATTTCGACACAACGTGCGCGCGCGGCCGAC : 2212
```

```
 -105                                                                                                      +1
R1 : GCACGATACCAAAGACAAACCCGCCAAAACGAGAAAAAAATAACTTATTGAGTGCCAGTGTGTGTTGTGTGCGTGATTGCGATTAGTGTTCTTTTTTTTTACAAAATGACGGC : 2329
R2 : GCACGATACCAAAGACAAACCCGCCAAAACGAGAAAAAAATAACTTATTGAGTGCCAGTGTGTGTTGTGTGCGTGATTGCGATTAGTGTT-TTTTTTTTTACAAAATGACGGC : 2321
S1 : GCACGATACCAAAGACAAACCCGCCAAAACGAGAAAAAAATTACTTATTGAGTGCCAGTGTGTGTTGTGTGCGTGATTGCGATTAGTGTT-TTTTTTTTTACAAAATGACGGC : 2300
S2 : GCACGATACCAAAGACAAACCCGCCAAAACGAGAAAAAAATTACTTATTGAGTGCCAGTGTGTGTTGTGTGCGTGATTGCGATTAGTGTT-TTTTTTTTTACAAAATGACGGC : 2310
S3 : GCACGATACCAAAGACAAACCCGCCAAAACGAGAAAAAAATTACTTATTGAGTGCCAGTGTGTGTTGTGTGCGTGATTGCGATTAGTGTT-TTTTTTTTTACAAAATGACGGC : 2302
S4 : GCACGATACCAAAGACAAACCCGCCAAAACGAGAAAAAAATTACTTATTGAGTGCCAGTGTGTGTTGTGTGCGTGATTGCGATTAGTGTT-TTTTTTTTTACAAAATGACGGC : 2336
S5 : GCACGATACCAAAGACAAACCCGCCAAAACGAGAAAAAAATAACTTATTGAGTGCCAGTGTGTGTTGTGTGCGTGATTGCGATTAGTGTT-TTTTTTTTTACAAAATGACGGC : 2330
S6 : GCACGATACCAAAGACAAACGCGCCAAAACGAGAAAAAAATAACTTATTGAGTGCCAGTGTGTGTTGTGTGCGTGATTGCGATTAGTGTT-TTTTTTTTTACAAAATGACGGC : 2324

R1 : GGGAAATGAGGAGCAGGAGCCTCTGAT : 2356
R2 : GGGAAATGAGGAGCAGGAGCCTCTGAT : 2348
S1 : GGGAAATGAGGAGCAGGAGCCTCTGAT : 2327
S2 : GGGAAATGAGGAGCAGGAGCCTCTGAT : 2337
S3 : GGGAAATGAGGAGCAGGAGCCTCTGAT : 2329
S4 : GGGAAATGAGGAGCAGGAGCCTCTGAT : 2363
S5 : GGGAAATGAGGAGCAGGAGCCTCTGAT : 2357
S6 : GGGAAATGAGGAGCAGGAGCCTCTGAT : 2351
```

**Figure S1.** Nucleotide sequence alignment of different 5′-UTRs of the *PxABCG1* gene in the Bt-susceptible DBM1Ac-S and Bt-resistant NIL-R strains. The nucleotides are numbered relative to the translation start site (ATG) indicated by +1, with the upstream sequence preceded by “-”.

| Promoters | R1 | R2 | S1 | S2 | S3 | S4 | S5 | S6 |
|---|---|---|---|---|---|---|---|---|
| R1 | 100% | | | | | | | |
| R2 | 96.63% | 100% | | | | | | |
| S1 | 96.66% | 97.11% | 100% | | | | | |
| S2 | 96.50% | 98.00% | 97.23% | 100% | | | | |
| S3 | 96.03% | 97.57% | 96.76% | 99.53% | 100% | | | |
| S4 | 95.27% | 96.67% | 95.90% | 98.10% | 97.63% | 100% | | |
| S5 | 94.65% | 95.50% | 95.77% | 95.41% | 95.11% | 97.22% | 100% | |
| S6 | 94.76% | 95.20% | 94.47% | 94.95% | 94.65% | 96.84% | 98.68% | 100% |
| | Group 1 | | | Group 2 | | Group 3 | | Group 4 |

| Sequence similarity | <96% | <98% | <100% | 100% |
|---|---|---|---|---|

**Figure S2.** Pairwise comparisons of sequence identities among different 5′-UTRs of the *PxABCG1* gene in the Bt-susceptible DBM1Ac-S and Bt-resistant NIL-R strains.

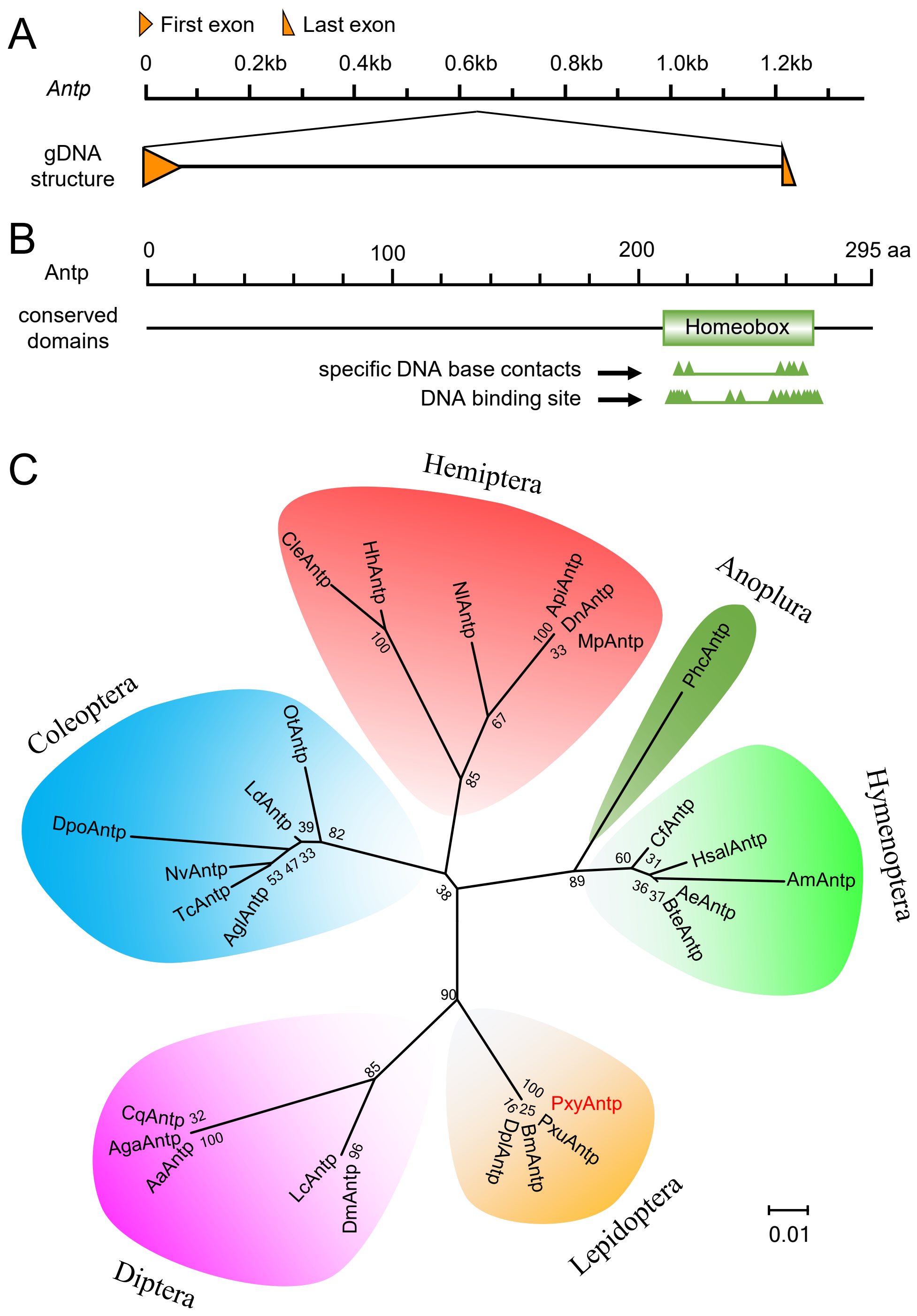


**Figure S3.** Structure and phylogenetic analyses of the *Antp* gene. (A) Genomic structure of the *Antp* gene in *P. xylostella*. Orange boxes denote the exons, and the space between two boxes denotes the intron. (B) Annotation of the conserved domains of the Antp protein based on the NCBI Conserved Domain Database (CDD). (C) Phylogenetic tree analysis of the Antp protein. The amino acid sequences of *Antp*

genes in different insects were retrieved from the GenBank database. Abbreviations: Abbreviations: **1. Lepidoptera** [**Bm** (*Bombyx mori*, XP_012549565); **Dpl** (*Danaus plexippus*, XP_032516004); **Pxy** (*Plutella xylostella*, XP_011555709); **Pxu** (*Papilio xuthus*, XP_013174066)]; **2. Anoplura** [**Phc** (*Pediculus humanus corporis*, XP_002426644)]; **3**. **Coleoptera** [**Agl** (*Anoplophora glabripennis*, XP_018561276); **Dpo** (*Dendroctonus ponderosae*, XP_019760912); **Ld** (*Leptinotarsa decemlineata*, XP_023018465); **Nv** (*Nicrophorus vespilloides*, XP_017772124); **Ot** (*Onthophagus taurus*, XP_022907641); **Tc** (*Tribolium castaneum*, AAK96031)]; **4. Diptera** [**Aa** (*Aedes aegypti*, XP_021694198); **Aga** (*Anopheles gambiae*, XP_311618); **Cq** (*Culex quinquefasciatus*, EDS27380); **Dm** (*Drosophila melanogaster*, NP_996166); **Lc** (*Lucilia cuprina*, XP_023300554)]; **5. Hymenoptera** [**Ae** (*Acromyrmex echinatior*, XP_011056342); **Am** (*Apis mellifera*, NP_001011571); **Bte** (*Bombus terrestris*, XP_003402194); **Hsal** (*Harpegnathos saltator*, XP_011148808); **Cf** (*Camponotus floridanus*, XP_011258060)]; **6. Hemiptera** [**Api** (*Acyrthosiphon pisum*, XP_029344361); **Cle** (*Cimex lectularius*, XP_014240210); **Dn** (*Diuraphis noxia*, XP_015367095); **Hh** (*Halyomorpha halys*, XP_014274854); **Mp** (*Myzus persicae*, XP_022180931); **Nl** (*Nilaparvata lugens*, XP_039289814)].

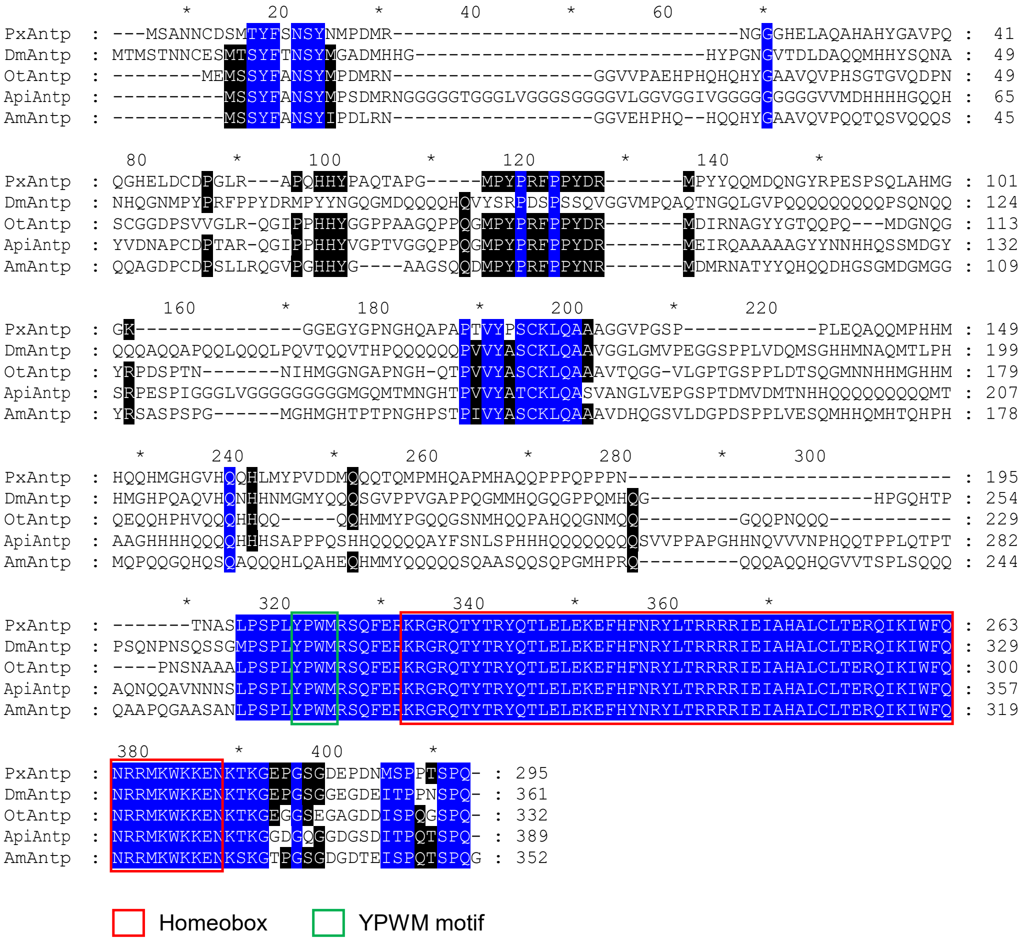


**Figure S4.** Multiple amino acid sequence alignment of Antp proteins in different insects. These proteins contain a conserved homeobox (indicated by red box), as well as a YPWM motif (indicated by green box) in the N-terminus adjacent to the homeobox. PxyAntp (*Plutella xylostella*, GenBank accession no. XP_011555709), DmAntp (*Drosophila melanogaster*, GenBank accession no. NP_996166), OtAntp (*Onthophagus taurus*, GenBank accession no. XP_022907641), ApiAntp (*Acyrthosiphon pisum*, GenBank accession no. XP_029344361), AmAntp (*Apis mellifera*, GenBank accession no. NP_001011571).

**Table S1.** Primers used for cloning *PxABCG1* promoter and construction of pGL4.10 recombinant plasmids.

| Purpose | Primer name | Primer sequence (5′-3′) | PCR product size (bp) | Positions |
|---|---|---|---|---|
| Promoter cloning | PxABCG1-F | ATGTCGATGTCGGTGGTTTAGC | 2313 | — |
| | PxABCG1-R | TTTGTAAAAAAAAAAACACTAATCG | | |
| Recombinant upstream† | P(-2313/-1)-F | ATGTCGATGTCGGTGGTTTAGC | 2313 | |
| | P(-1777/-1)-F | ATAATTATGAAACAGTCTACTTACAG | 1777 | -2313/-1 |
| | P(-1307/-1)-F | GGACAAAACACATGTAGGTATCATC | 1307 | -1777/-1 |
| | P(-1186/-1)-F | AAAAGCTTCCTATGTTTGTG | 1186 | -1186/-1 |
| | P(-1107/-1)-F | GTGCAATGTAAATAGATACCATT | 1107 | -1107/-1 |
| | P(-972/-1)-F | ACGTATACCTAAATACGCTTT | 972 | -972/-1 |
| | P(-891/-1)-F | ACGGTCTTAGTTAAAACGCT | 891 | -891/-1 |
| | P(-806/-1)-F | TAAGAAAACATACTTAGTAAGTACC | 806 | -806/-1 |
| | P(-310/-1)-F | TAATAACTGTCTCTTTGTAACAA | 310 | -310/-1 |
| Recombinant downstream‡ | Promoter-R | TTTGTAAAAAAAAAAACACTAATCG | — | — |

†A 15 bp sequence (TGGCCTAACTGGCCG) is added to the 5′ ends of the forward (F) primers for construction of pGL4.10 recombinants containing *PxABCG1* promoter.

‡ A 15 bp sequence (CGCCGAGGCCAGATC) is added to the 5′ ends of the reverse (R) primer for construction of pGL4.10 recombinants containing *PxABCG1* promoter.

**Table S2.** Primers used for transcription factor (TF) cloning and construction of recombinant plasmids.

| Purpose | Primer name | Primer sequence (5′-3′) | PCR product size (bp) | Positions (bp) |
|---|---|---|---|---|
| TF cloning | Antp-F | GTGAATGGAATCCGAAGTGT | 1054 | -112–942 |
| | Antp-R | ACGGGTAAACCTGAAACAAT | | |
| | Dfd-F | GCTCAGTCGTTGATTCGC | 1241 | -62–1179 |
| | Dfd-R | GTTTGCAACATTGTTTAAAGAT | | |
| pAc5.1-TF† | In-Antp-F | ATGAGCGCCAACAACTGCGA | 885 | 1–885 |
| | In-Antp-R | TTGTGGCGAGGTGGGCG | | |
| | In-Dfd-F | ATGCGCGACCCCGCCCC | 1155 | 1–1155 |
| | In-Dfd-R | TAAGGCGGTGAGACCGTAGT | | |
| pGADT7-PxAntp‡ | Antp-Y1H-F | ATGAGCGCCAACAACTGCGA | 885 | 1–885 |
| | Antp-Y1H-R | TTGTGGCGAGGTGGGCG | | |

† A 15 bp sequence (AGACCCCGGATCGGG) is added to 5′ ends of forward primers of pAc5.1-TF recombinant plasmids; A 15 bp sequence (GCCCTCTAGACTCGA) is add to 5′ ends of reverse primers of pAc5.1-TF recombinant plasmids.

‡ A 15 bp sequence (GGAGGCCAGTGAATT) is added to the 5′ end of forward primer of pGADT7-Antp recombinant plasmid; A 15 bp sequence (TCATCTGCAGCTCGA) is added to the 5′ end of the reverse primer of pGADT7-Antp recombinant plasmid.

**Table S3.** Primers used for qPCR and dsRNA synthesis.

| Purpose | Primer name | Primer sequence (5′-3′) | PCR product size (bp) | Positions (bp) |
|---|---|---|---|---|
| qPCR analysis | qAntp-F | AACTCGTACAACATGCCGGA | 140 | 40–179 |
| | qAntp-R | GGGTAGTGGTGCTGCGG | | |
| | qABCG1-F | ATCTGGTGTTTCAGGCTTTAGTC | 118 | 545–662 |
| | qABCG1-R | ATCACGGTGTTCTGGCATTT | | |
| | qL32-F | CCAATTTACCGCCCTACC | 120 | — |
| | qL32-R | TACCCTGTTGTCAATACCTCT | | |
| dsRNA synthesis† | dsAntp-F | T7-ATGCCGTACTACCAGCAGATG | 278 | 232-509 |
| | dsAntp-R | T7-ATGTCGTCCACCGGGTACAT | | |
| | dsEGFP-F | T7-CCACAAGTTCAGCGTGTCCG | 469 | — |
| | dsEGFP-R | T7-AAGTTCACCTTGATGCCGTTC | | |

†The T7 RNA polymerase promoter sequence (TAATACGACTCACTATAGGGAGA) is added to 5′ ends of forward and reverse primers to synthesize dsRNA template.
